# External validity of rct evidence in cost-effectiveness analyses. A review of recent technology appraisals for nice and proposed methods of adjustment

**DOI:** 10.1186/1745-6215-16-S2-P25

**Published:** 2015-11-16

**Authors:** Theodoros Mantopoulos, Sofia Dias, Antony Ades, Nicky Welton

**Affiliations:** 1 University of Bristol, Bristol, UK

## Introduction

Randomised Controlled Trials (RCTs) produce relative treatment effects that have high internal validity. However they may suffer from lack of external validity when used as inputs to cost-effectiveness analyses (CEA). If differences in RCT and CEA populations can be described by a set of observed covariates, then it is possible to adjust for these differences. A common approach is to estimate effects at the mean covariate values. For non-linear models this introduces bias, even if the RCT and CEA populations are comparable.

## Objectives

(i) To review recent technology appraisals (TAs) submitted to NICE to see how covariate adjustment in CEA is typically conducted and (ii) to explore the impact that failing to appropriately adjust for covariates can have on model outputs.

## Methods

We reviewed all TAs issued by NICE in 2014. We explore, for binary outcomes, when the bias from using the covariate mean approach is likely to have an impact.

## Results

7/25(28%) reported important differences between RCT and CEA populations, and only 4 of those 7 adjusted for covariates in the CEA model. 17 of the 25 TAs adjusted for covariates, of which 3 used the mean of the covariates, 2 averaged over the covariate distribution, and 8 performed subgroup analyses, and the method of adjustment was unclear in 4. The bias introduced by using the mean of the covariates can be large and increases with the variance in the covariate distribution.

## Conclusions

Covariate adjustment requires integration over the joint covariate distribution in the CEA population.

